# Novel Fabrication of Silver-Coated Copper Nanowires with Organic Compound Solution

**DOI:** 10.3390/ma15031135

**Published:** 2022-02-01

**Authors:** Suhyun Lee, Chien Wern, Sung Yi

**Affiliations:** Department of Mechanical and Materials Engineering, Portland State University, Portland, OR 97207-075, USA; suhyun@pdx.edu (S.L.); wernc@pdx.edu (C.W.)

**Keywords:** synthesis, nanowires, copper nanowires, silver coating, core–shell nanowires, Cu-Ag nanowires

## Abstract

Copper nanowires and Cu-Ag nanowires have various potential applications, such as transparent conductive film, flexible electronics, and conductive filler. In this study, we developed a new green fabrication method for silver-coated copper nanowires using methylsulfonylmethane (DMSO_2_), which is an environmentally friendly chemical at the food-grade level, to replace toxic chemicals, including ammonia, in the silver coating process. Copper nanowires were synthesized under various reaction temperatures and concentrations of hydrazine (N_2_H_4_), ethylenediamine (EDA), sodium hydroxide (NaOH), and copper precursor. The reaction temperature higher than 70 °C caused the oxidation of copper products and evaporation of the sample solution. The optimal conditions to synthesize copper nanowires more than 18 µm in length and 25–45 nm in diameter were determined: 9 M of NaOH, 50 µL of EDA, 17 mM of CuCl_2_, 5.7 mM of N_2_H_4_, and 70 °C reaction temperature. Cu-Ag nanowires, which have about a 12 nm thick silver shell, were successfully fabricated at room temperature under 1 mM of silver nitrate (AgNO_3_) and 1 wt % of DMSO_2_. Synthesis conditions for copper and silver-coated copper nanowires have been optimized.

## 1. Introduction

Transparent electrodes are essential components for flat-panel displays, touch screens, solar cells, and flexible electronics such as foldable tablets and phones, bendable lighting emitting diodes (LEDs), and wearable sensors. Indium tin oxide (ITO) is widely used to fabricate transparent conductors because of its high conductivity, low resistance, and high transmittance [1]. However, ITO is brittle, expensive, and has poor reserves on Earth [2]. When ITO is used on flexible electronics, the substrate can crack when bending or stretching is applied. In addition, it is difficult to produce at a low cost and large scale because of its scarcity [3]. 

To overcome the limitations of this conventional material, the synthesis of 2D copper nanosheets has intensively been researched in previous work [4]. Several other nanomaterials, including 1D carbon nanotubes (CNTs), graphene, and 1D metal nanowires, have also been considered as alternatives [5,6]. Those nanomaterials can achieve flexibility; however, the sheet resistance of CNTs (150 Ω/sq) and graphene (250 Ω/sq) is higher than that of ITO (20 Ω/sq) [7,8]. Because of their higher sheet resistance, metal nanowires are considered promising alternative materials. Among various metal nanowires, the sheet resistance of silver (10 Ω/sq) and copper (34.8 Ω/sq) nanowires meets the property required to be comparable with ITO [9]. Copper nanowires receive more attention than silver nanowires. This is because copper not only has the second-highest conductivity and lowest resistivity, but also has extremely high reserves [10,11,12]. In addition, the stretchable reliability of copper nanowires has been demonstrated through the test of repeated stretching and releasing on wearable stretchable sensors, showing current stability and high sensitivity [6].

To synthesize copper nanowires, researchers have attempted several methods, such as chemical vapor deposition [13,14], vacuum thermal decomposition [15], the template-based method [16,17], and the solution-based method [18,19]. The chemical vapor deposition method and vacuum thermal decomposition method require a processing temperature over 200 °C, and the template-based method requires complex electrode preparation. On the other hand, the solution-based method has a simple procedure with a relatively low processing temperature compared to chemical vapor deposition and vacuum thermal decomposition. Moreover, it is possible to control the quality of copper nanowires during the reaction time [20].

The solution-based method is mainly carried out by controlling the number of copper precursor ions to obtain pure copper nanowires. The synthesis of copper nanowires by the solution-based method consists of a reagent, copper precursor, capping agent, and reducing agent. Li et al. [21] synthesized copper nanowires using oleylamine and glucose as a capping agent and a reducing agent, respectively, with the length of 60–90 µm and the diameter of 45 ± 3 nm. However, it required a long reaction time of 12 h and a high reaction temperature of 116 °C. In addition, using oleylamine as a capping agent requires combining it with other capping agents such as oleic acid and potassium bromide (KBr) [22]. Chang et al. [18] researched the synthesis of copper nanowires by using ethylenediamine (EDA) as a capping agent and hydrazine (N_2_H_4_) as a reducing agent. They obtained copper nanowires that are 40–50 µm in length and 90–120 nm in diameter at 60 °C. Rathmell et al. [19] implemented the approach based on Chang et al. [18] at a larger scale to obtain 1.2 g of copper nanowires having the length of 10 ± 3 µm and the diameter of 90 ± 10 nm at the processing temperature of 80 °C for 1 h. Such copper nanowires were grown from spherical nanoparticles found at the end of copper nanowires. Koo et al. [23] demonstrated the relationship between EDA and copper nanowire oxidation. EDA plays a role in reducing hydroxide adsorption, which increases the resistivity of copper nanowire oxidation. 

Copper nanowires are very vulnerable to contact with oxygen, which causes the formation of an oxide layer on the surface and a subsequent reduction in electrical conductivity [24]. In addition, the irregular surface of the copper nanowires is susceptible to defects, which would cause dangling chemical bonds on the surface of the copper nanowires. This chemical bond results in the oxidation of the copper nanowires, reducing surface energy [25]. The formation of an oxidation-resistant metallic shell on the surface of the copper nanowires is considered an effective way to enhance oxidation resistance. Among metals, nickel (Ni), platinum (Pt), gold (Au), and silver (Ag) are suitable shell materials because of their low resistivity and high electrical conductivity. In particular, if silver is used as a shell material and silver oxide is formed on the surface of copper nanowires, it has much higher electrical conductivity than copper oxide [26].

To fabricate core–shell nanowires, the electroplating method and the galvanic replacement method are generally used. The electroplating method prevents metal shell deposition from aggregating and is used for a wide range of metals including Ni, Pt, and Au. However, this method has low efficiency as it does not uniformly plate the entire copper nanowires [27]. The galvanic replacement method provides simple and diverse multifunctional nanostructures. However, breakage of copper nanowires can be caused when plating a metal with a higher reduction potential than copper due to corrosion and oxidation [28].

Niu et al. [29] conducted the fabrication of Cu-Au nanowires through the growth of the atomic layer of Au precursor. Chen et al. [30] used the electroplating method to obtain Cu-Pt nanowires. Those nanowires demonstrated oxidation stability under the ambient condition of long-period exposure. However, Au and Pt are precious and expensive metals, so these nanowires are expensive. Luo et al. [26] and Jiang et al. [31] developed well-dispersed silver nanoparticles on the surface of copper nanowires using the galvanic replacement method in the presence of ammonium hydroxide (NH_4_OH). Dissolved silver in the NH_4_OH solution reacted with the copper atom that brought about the deposition of silver nanoparticles on the surface of copper nanowires. The weight of Cu-Ag nanowires increased at a higher temperature exposure than copper nanowires, and the total increasing rate of Cu-Ag nanowires was about eight times higher than that of copper nanowires [26]. While the sheet resistance of the copper nanowire film exposed at 160 °C increased rapidly within a few hours, the sheet resistance of the Cu-Ag nanowire film was stable for more than 1 day, with no significant difference compared with silver nanowires [32]. Furthermore, a 5 nm thick silver shell exhibited a similar sheet resistance value as silver nanowires at 160 °C for 24 h. The oxidation of copper nanowires could not be prevented at 85 °C/85% relative humidity, whereas the 15 nm thick silver shell copper nanowire was stable [33]. 

The present study is focused on developing the fabrication of silver-coated copper nanowires and a preparation method to replace ammonia through an electroless solution-based method. A new green fabrication method for Cu-Ag nanowires is developed using DMSO_2_, which is an environmentally friendly chemical at the food-grade level, to substitute ammonia in the silver coating process. DMSO_2_ has not been reported previously for the fabrication of core–shell nanowires. The effects of different reaction temperatures and concentrations of N_2_H_4_, EDA, NaOH, and copper precursor on the morphology of copper nanowires are compared and analyzed. In addition, silver nitrate (AgNO_3_) is employed as a shell material to coat the surface of copper nanowires. Copper nanowires and Cu-Ag nanowires are characterized by scanning electron microscopy (SEM) and transmission electron microscopy (TEM). 

## 2. Materials and Methods

### 2.1. Materials

To synthesize copper nanowires, a reagent, copper precursor, a capping agent, and a reducing agent are required. Sodium hydroxide (NaOH) was employed as a reagent to decompose copper precursors at the beginning of the procedure. Copper chloride (CuCl_2_) was utilized as a copper precursor. Ethylenediamine (EDA, C_2_H_8_N_2_, 99.5%, Sigma-Aldrich, Burlington, MA, USA) and hydrazine (N_2_H_4_, 35 wt % in H_2_O, Sigma-Aldrich, Burlington, MA, USA) were used as a capping agent and a reducing agent, respectively. To fabricate Cu-Ag nanowires, silver nitrate (AgNO_3_) was consumed as aa shell material to coat on the surface of copper nanowires, and methylsulfonylmethane (DMSO_2_, >99.8%, Bergstrom Nutrition, Vancouver, WA, USA) was used as a copper surfactant and a silver reducing agent. Deionized water (DI H_2_O) was used as a solvent to dissolve NaOH, CuCl_2_, AgNO_3_, and DMSO_2_ and to dilute N_2_H_4_. 

### 2.2. Method for Synthesis of Copper Nanowires

The solution-based method was employed to synthesize copper nanowire. All experiments were carried out under 300 rpm magnetic stirring to reach a homogenous state. An Erlenmeyer flask with the material solution was placed in the water bath to keep the constant reaction temperature (40, 50, 60, 70, 80, 90 °C) for 2 h reaction time. NaOH (4.8, 5, 7, 9, 12, and 14.7 M), CuCl_2_ (17 and 37 mM), and N_2_H_4_ (1.7 and 5.7 mM) solutions were prepared by dissolving with DI H_2_O separately. First, NaOH was dissolved in DI H_2_O under magnetic stirring for 10 min. EDA was then added to NaOH solution under magnetic stirring for 2 min. In this process, the solution is kept colorless. At the end of 2 min, CuCl_2_ solution was added to the colorless solution. The addition of CuCl_2_ solution turned light blue and then dark blue at the end of 10 min due to CuCl_2_ decomposition, as shown in Equation (1):

2NaOH (aq) + CuCl_2_ (aq) → Cu(OH)_2_ (aq) + 2NaCl (1)

At the end of 10 min, N_2_H_4_ was added to the dark blue solution. Copper hydroxide (Cu(OH)_2_) was decomposed to copper oxide (Cu_2_O) by adding N_2_H_4_ through Equation (2), and Cu_2_O nanoparticles were further reduced to copper seeds through Equation (3):

Cu(OH)_2_ (aq)+ N_2_H_4_ (aq) → 2Cu_2_O (s) + H_2_O (2)

2Cu_2_O (s) + N_2_H_4_ (aq) → 4Cu (s) + N_2_ (g) + 2H_2_O (3)

Copper seeds produced in Equation (3) then grew into copper nanowires with continuous heating under magnetic stirring. As soon as N_2_H_4_ was added, the dark blue solution turned white and then turned reddish-brown after 2 h of reaction time. At the end of 2 h of stirring, the copper nanowire solution was washed with methanol to eliminate chemical impurities and dried in a vacuum desiccator at room temperature for at least 2 h.

### 2.3. Method for Fabrication of Cu-Ag Nanowires

Cu-Ag nanowires were fabricated by a new green method, adding DMSO_2_ at room temperature without electrodes and heating. This process was conducted under 300 rpm magnetic stirring to reach a homogenous state. First, 1 wt % of DMSO_2_ and 1 mM of AgNO_3_ solution were prepared in an Erlenmeyer flask separately under magnetic stirring. Then, 1 wt % of DMSO_2_ was added to well-dispersed copper nanowire solution (from the previous section) under magnetic stirring for 5 min, and then AgNO_3_ solution was added at the end of 5 min. When AgNO_3_ solution was added, the solution gradually turned from reddish-brown to gray. During the 10 min reaction time, the solution turned dark gray. At the end of 10 min, the Cu-Ag nanowire solution was washed with methanol and dried in a vacuum desiccator at room temperature for at least 2 h. 

### 2.4. Characterization

To characterize bulk copper nanowires and Cu-Ag nanowires, scanning electron microscopy (SEM, Helios 400, FEI, Hillsboro, OR, USA) with an accelerating voltage of 5 kV was utilized. The morphology and dimensions including the length and diameter of copper nanowires and Cu-Ag nanowires were observed by transmission electron microscopy (TEM, Tecnai F-20, FEI, Hillsboro, OR, USA) with an accelerating voltage of 200 kV. Energy-dispersive X-ray spectroscopy (EDX)was used for the elemental analysis of Cu-Ag nanowires.

## 3. Results and Discussion

### 3.1. The Effect of Concentration of N_2_H_4_

**Figure 1:**
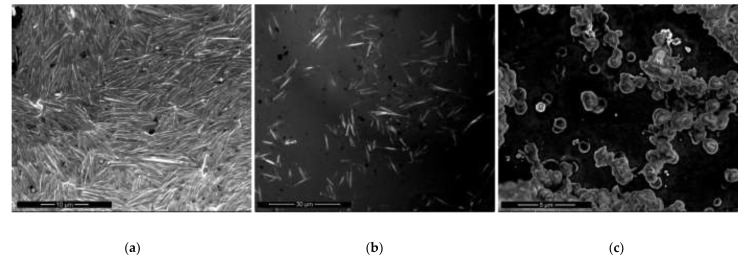
SEM images of copper nanowires with: (**a**) 15 µL; (**b**) 30 µL; (**c**) 240 µL of N_2_H_4_.

The concentrations of NaOH, EDA, and CuCl_2_ were held constant throughout this study, and the reaction temperature and time were kept at 60 °C and 2 h, respectively. Copper nanowires were synthesized with different amounts of 1.7 mM of N_2_H_4_. Figure 1 indicates the dimension distribution, including length and diameter. 

By adding 15 µL of N_2_H_4_, the synthesized copper nanowires were 4–13 µm in length and 250–550 nm in diameter, as shown in Figure 1a. Meanwhile, copper nanowires synthesized with 30 µL of N_2_H_4_ were 4–15 µm in length and 450–750 nm in diameter, as shown in Figure 1b. There are no significant differences in dimensions between copper nanowires synthesized with 15 µL and 30 µL of N_2_H_4_. However, a higher yield of copper nanowires is shown in Figure 1a compared to Figure 1b. Interestingly, copper nanoparticles, which are 800 nm in diameter, are observed dominantly without copper nanowires, as shown in Figure 1c. Since N_2_H_4_ is a strong reducing agent, the additional amount of N_2_H_4_ increases the diameter of the copper nanowires. However, an excessive amount of N_2_H_4_ causes an imbalance in the process of growing copper nanowires from copper seeds. Therefore, the process may not be completely conducted exhibiting the dominant copper nanoparticles. Table 1 summarizes the dimensions of length and diameter for each copper product synthesized with various amounts of N_2_H_4_. 

### 3.2. The Effect of Temperature on Morphology of Copper Nanowires

**Figure 2:**
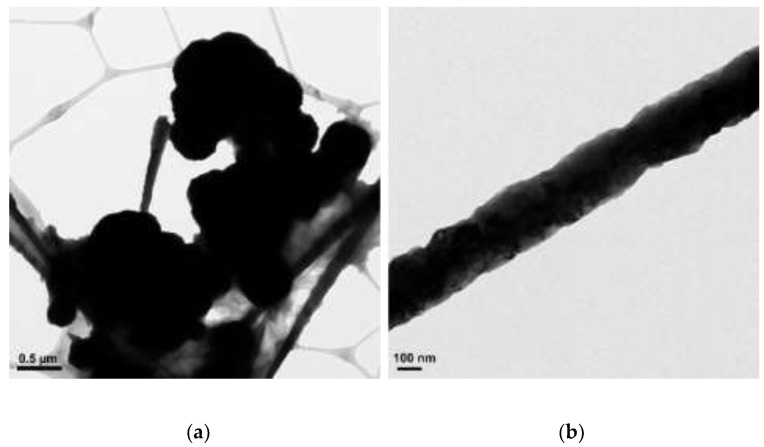
TEM images of copper products at 40 °C: (**a**) scale bar 0.5 µm; (**b**) scale bar 100 nm.

**Figure 3:**
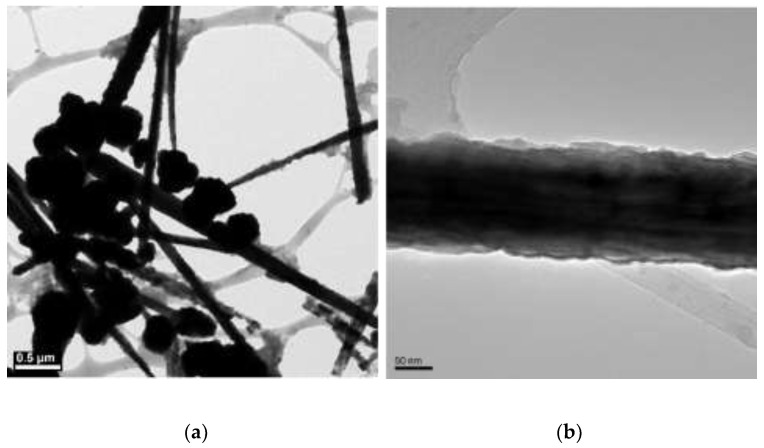
TEM images of copper products at 50 °C: (**a**) scale bar 0.5 µm; (**b**) scale bar 50 nm.

**Figure 4:**
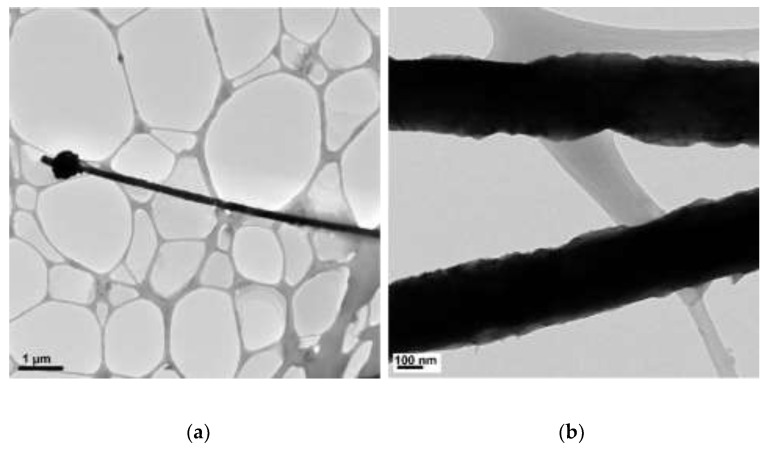
TEM images of copper products at 60 °C: (**a**) scale bar 1 µm; (**b**) scale bar 100 nm.

**Figure 5:**
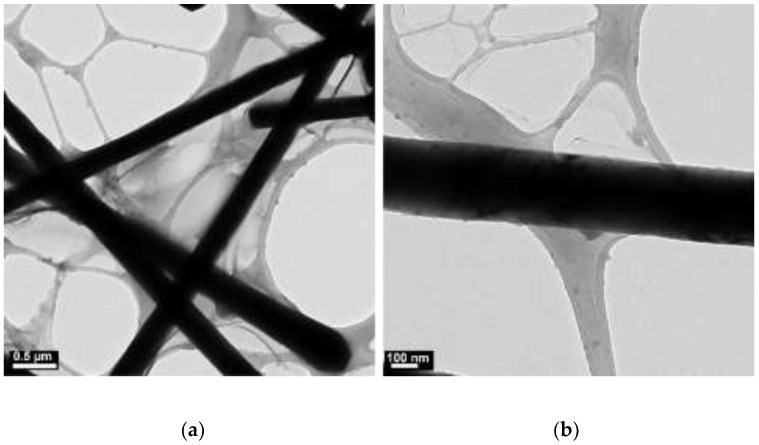
TEM images of copper products at 70 °C: (**a**) scale bar 0.5 µm; (**b**) scale bar 100 nm.

**Figure 6:**
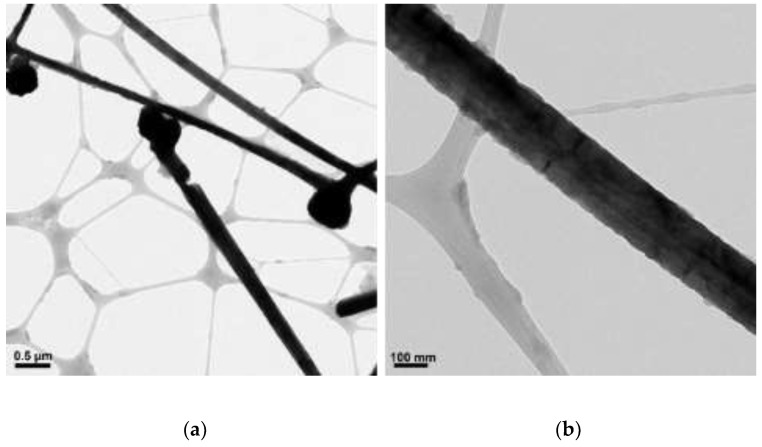
TEM images of copper products at 80 °C: (**a**) scale bar 0.5 µm; (**b**) scale bar 100 nm.

**Figure 7:**
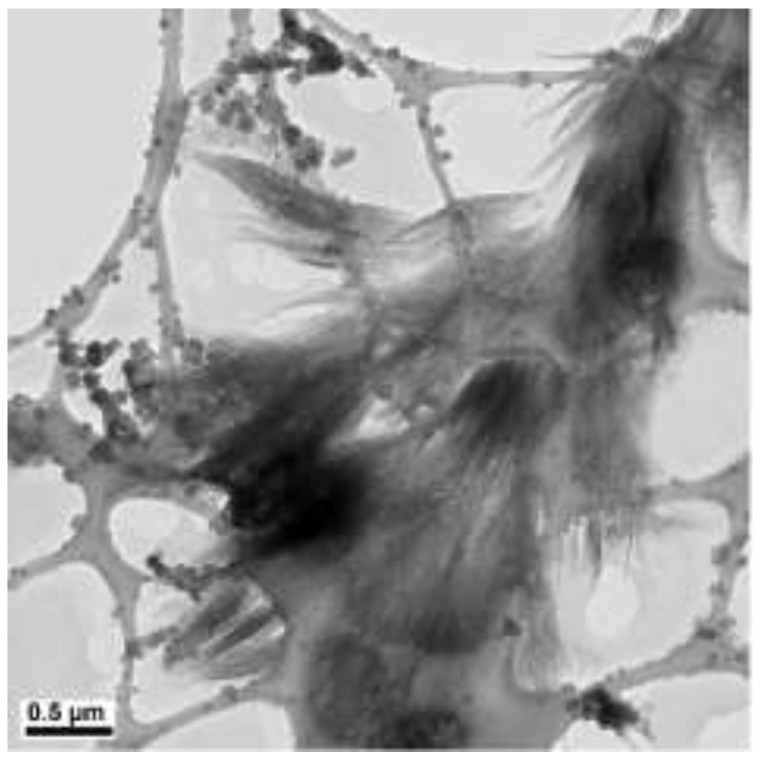
TEM images of copper products at 90 °C (scale bar 0.5 µm).

The concentrations of 4.8 M of NaOH, 30 µL of EDA, 17 mM of CuCl_2_, and 5.7 mM of N_2_H_4_ were held constant, and various reaction temperatures (40, 50, 60, 70, 80, and 90 °C) were maintained for a 2 h reaction time. Figure 2, Figure 3, Figure 4, Figure 5, Figure 6 and Figure 7 show copper nanowires synthesized at various reaction temperatures that have different morphology and dimensions. As shown in Figure 2a, copper nanowires synthesized at 40 °C show the aggregation of copper products, which makes it difficult to grow copper nanowires. In addition, the surfaces are not smooth, with some dented areas in Figure 2b. Copper nanowires measure more than 3.5 µm in length and 130–250 nm in diameter. The aggregation of copper products is slightly reduced at a higher reaction temperature of 50 °C, but the surfaces of copper nanowires are still rough, as shown in Figure 3. Copper nanowires synthesized at 50 °C measure more than 5 µm in length and 130–280 nm in diameter. Figure 4a shows that there is a greater reduction in copper aggregation synthesized at 60 °C than 40 or 50 °C. In addition, Figure 4b shows that the copper nanowires measure more than 8 µm in length and 200–310 nm in diameter. Interestingly, as the reaction temperature is increased to 70 °C, the smoothness of the surface is significantly improved without the aggregation of copper products, as shown in Figure 5. Copper nanowires synthesized at 70 °C measure more than 5 µm in length and 210–260 nm in diameter. 

Copper nanowires synthesized at 80 °C measure more than 6 µm in length and 120–280 nm in diameter. At this temperature, copper nanowires start to have kinks at the end, as shown in Figure 6a. The kinks may degrade the physical properties of the copper nanowires, including electrical and thermal conductivity, since there is a reduction in the cross-sectional area due to the kinks. With a further increased reaction temperature to 90 °C, copper products display oxidation, as shown in Figure 7. This is because the oxidation of copper is accelerated by a higher reaction temperature. 

Table 2 summarizes the dimensions of the copper nanowires synthesized at various temperatures. It can be seen that there is no tendency to become greater in length and thinner in diameter as the reaction temperature is increased. However, the reaction temperature affects the formation of copper seeds and growth of copper nanowires. 

Based on the aggregation of copper products and the morphology of copper nanowires, EDA as a capping agent is affected by the reaction temperatures since the capping agent prevents precipitation and aggregation by protecting the surface of copper seeds. To protect the surface of copper seeds, the reaction temperature of at least 60 °C, at which the aggregations are not observed, is required. However, EDA may perform actively at 70 °C to obtain a smooth surface without the aggregation of the copper products by providing sufficient surface energy.

### 3.3. The Effect of Concentration of EDA

**Figure 8:**
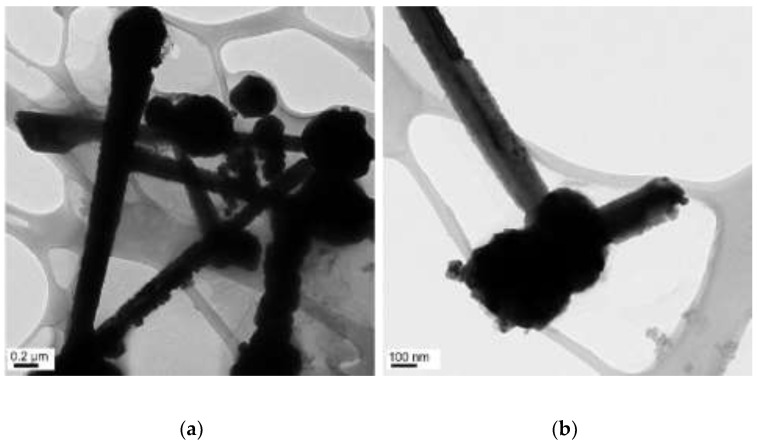
TEM images of copper nanowires with 15 µL of EDA: (**a**) scale bar 0.2 µm; (**b**) scale bar 100 nm.

**Figure 9:**
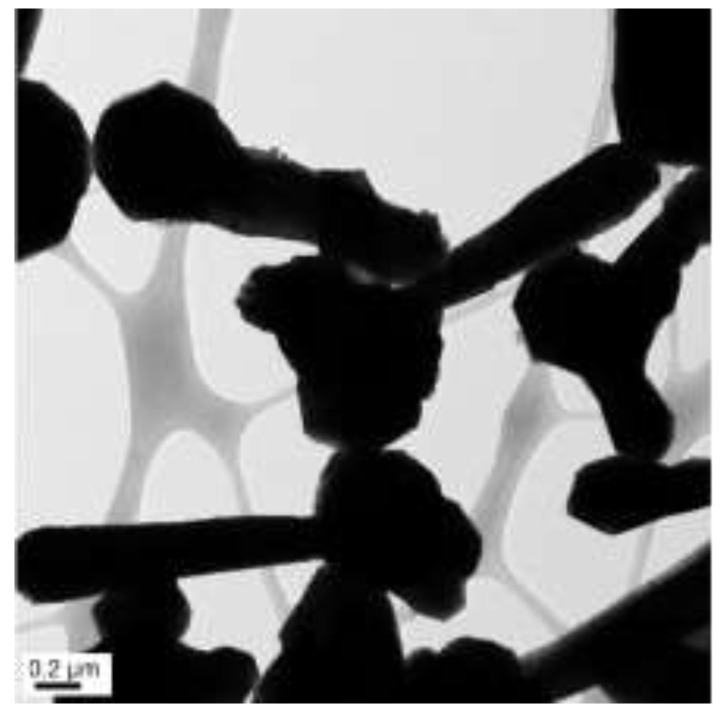
TEM images of copper nanowires with 50 µL of EDA (scale bar 0.2 µm).

**Figure 10:**
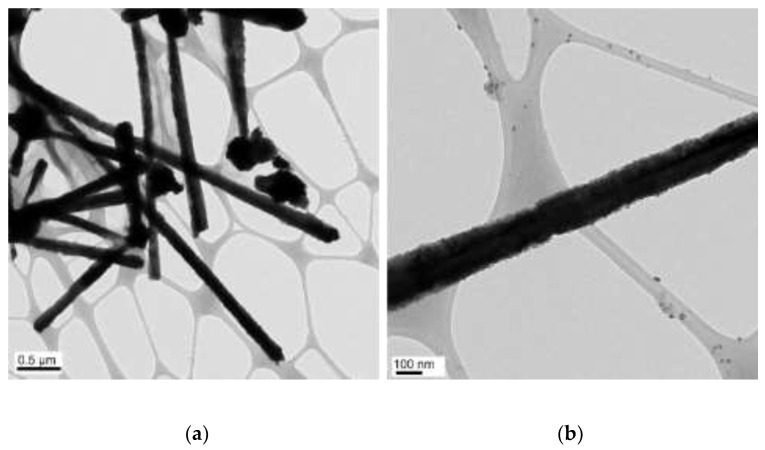
TEM images of copper nanowires with 50 µL of EDA: (**a**) scale bar 0.5 µm; (**b**) scale bar 100 nm.

The concentrations of 14.7 M of NaOH, 17 mM of CuCl_2_, and 5.7 mM of N_2_H_4_ were held constant, and the reaction temperature and time were kept at 70 °C and 2 h, respectively. Copper nanowires were formed at various concentrations of EDA (15, 50, and 60 µL), as shown in Figure 8, Figure 9 and Figure 10. Copper nanowires synthesized by adding 15 µL of EDA measure more than 4 µm in length and 250–550 nm in diameter, as shown in Figure 8. A few copper seeds and irregular surfaces of copper nanowires are observed. The increased amount of EDA to 50 µL dominantly forms the tapered copper nanowires, as shown in Figure 9. The tapered copper nanowires could result from the unstably protected surface of copper seeds that makes it difficult to grow copper nanowires. Copper nanowires synthesized with 50 µL of EDA measure more than 2 µm in length and 210–270 nm in diameter. However, there are no copper seeds, and the surface of copper nanowires is quite smooth. With the further increased amount of EDA to 60 µL, the synthesized copper nanowires measure more than 6 µm in length and 130–170 µm in diameter. However, very small nanoparticles are observed on the surface of copper nanowires, as shown in Figure 10. The surface and morphology of copper nanowires are affected by the amounts of EDA. The addition of EDA does not guarantee the protection of the surface of copper seeds completely. However, EDA may control the dimensions of copper nanowires depending on the amount. An excessive amount of EDA erratically protects copper seeds, resulting in the irregular surface of copper nanowires. The irregular surface can generate defects in copper nanowires, including a dangling bond that is significantly sensitive to ambient conditions where copper nanowires can be oxidized. Table 3 summarizes the size distribution for each set of copper nanowires synthesized under various amounts of EDA. 

### 3.4. The Effect of Concentration of NaOH

**Figure 11:**
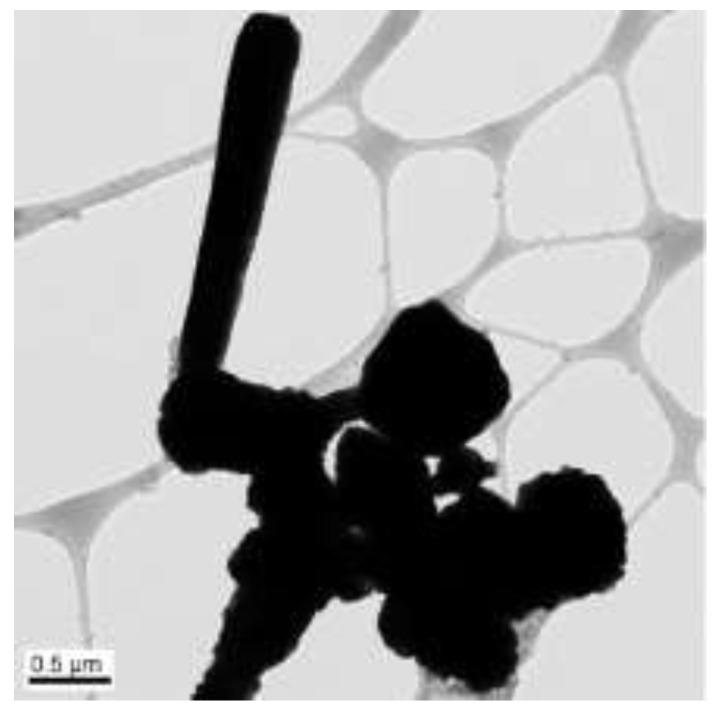
TEM images of copper nanowires with 5 M of NaOH (scale bar 0.5 µm).

**Figure 12:**
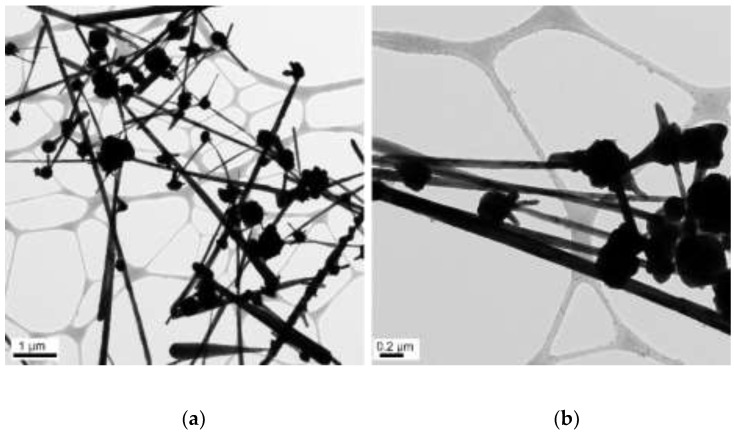
TEM images of copper nanowires with 7 M of NaOH: (**a**) scale bar 1 µm; (**b**) scale bar 0.2 µm.

**Figure 13:**
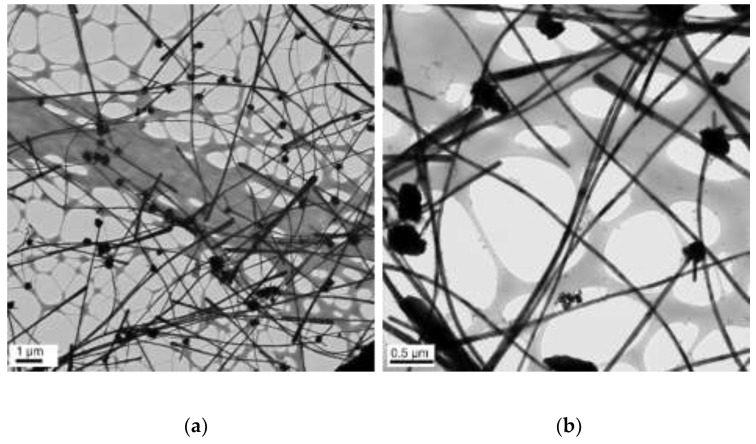
TEM images of copper nanowires with 9 M of NaOH: (**a**) scale bar 1 µm; (**b**) scale bar 0.5 µm.

**Figure 14:**
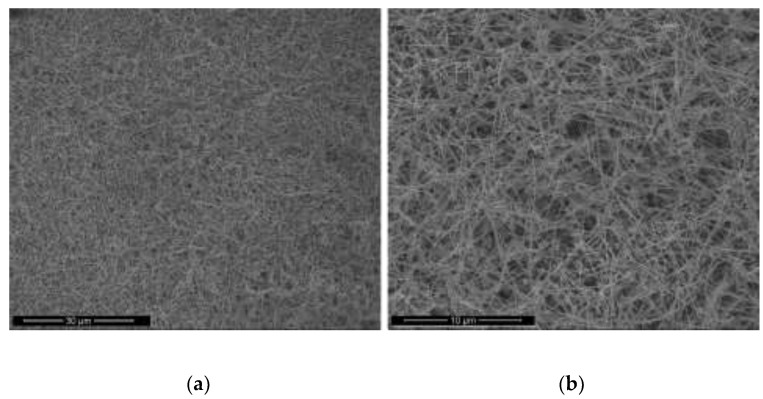
SEM images of copper nanowires with 9 M of NaOH: (**a**) scale bar 30 µm; (**b**) scale bar 10 µm.

**Figure 15:**
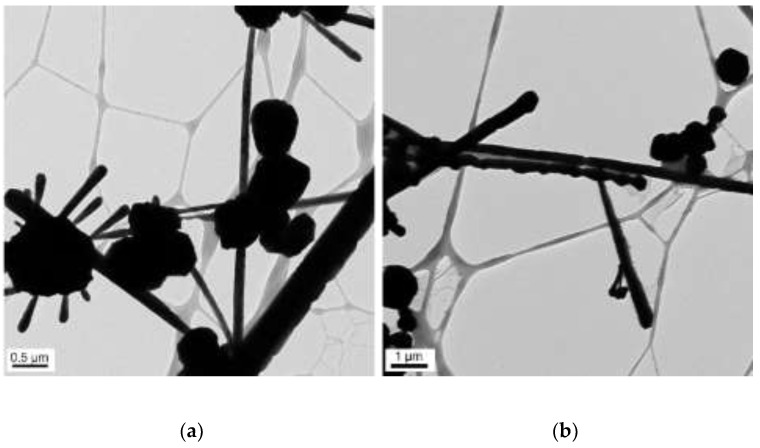
TEM images of copper nanowires with 12 M of NaOH: (**a**) scale bar 0.5 µm; (**b**) scale bar 1 µm.

The concentrations of 50 µL of EDA, 17.1 mM of CuCl_2_, and 5.7 mM of N_2_H_4_ were held constant, and the reaction temperature and time were preserved at 70 °C and 2 h, respectively. Copper nanowires were synthesized under various concentrations of NaOH (5, 7, 9, and 12 M). Figure 11 shows the copper nanowires synthesized with 5 M NaOH, indicating similar morphology as Figure 9, which shows copper wires synthesized with 14.7 M of NaOH. Copper nanowires more than 3 µm in length and 130–250 nm in diameter are observed in Figure 11. As shown in Figure 12, synthesized copper nanowires more than 12 µm in length and 80–160 nm in diameter are developed with a higher concentration of NaOH. However, there are some aggregations of copper seeds. The ultra-long and ultra-thin copper nanowires synthesized with 9 M of NaOH measure more than 18 µm in length and 25–45 nm in diameter, as shown in Figure 13. The dimensions are remarkably developed compared to 5 and 7 M of NaOH, as shown in Figure 11 and Figure 12, respectively. Copper nanowires begin to float on the top of the solution due to the high density of the solution without the centrifugation process. Moreover, a high yield of copper nanowires is obtained, as shown in Figure 14. The further increased concentration of NaOH to 12 M indicates the aggregation of copper seeds and a fivefold difference in the diameter, as shown in Figure 15.

The high concentration of NaOH above 9 M causes the incomplete formation of copper nanowires. This is because a high concentration of NaOH could not be completely dissolved in DI H_2_O, and the remaining NaOH may prevent copper seeds from being protected by EDA. The low concentration of NaOH below 9 M is likely required to increase the amount of EDA to improve the aggregation of copper seeds. This means that NaOH interplays with EDA. Therefore, the complete formation of copper nanowires can be affected by controlling the concentration of NaOH and EDA simultaneously. Table 4 summarizes the dimensions of copper nanowires. 

### 3.5. The Effect of Concentration of Copper Precursor

**Figure 16:**
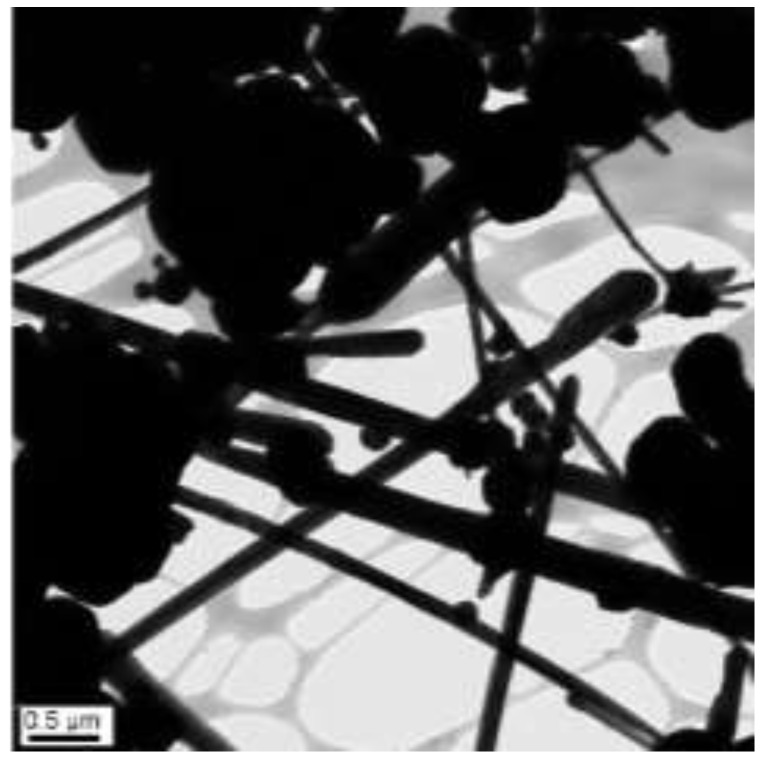
TEM image of copper nanowires with 37 mM of copper precursor (scale bar 0.5 µm).

The concentrations of 9 M of NaOH, 50 µL of EDA, and 5.7 mM of N_2_H_4_ were held constant, and the reaction temperature and time were 70 °C and 2 h, respectively. Copper nanowires were synthesized by adding the concentration of 37 mM of CuCl_2_. Compared to Figure 13, doubling the concentration of CuCl_2_ produces thicker and shorter nanowires with dimensions more than 6 µm in length and 100–240 nm in diameter. In addition, the increased concentration of copper precursor causes large aggregations of copper seeds, as shown in Figure 16. This is because EDA cannot protect the surface of copper seeds. These copper seeds may begin to agglomerate and make it difficult to grow copper nanowires. Therefore, the amount of EDA must be adjusted depending on the concentration of copper precursor. 

### 3.6. Fabrication of Cu-Ag Nanowires

Silver coating on the surface of copper nanowires is induced by a redox reaction between copper and silver through Equation (4):

Cu → Cu^2+^ + 2e^−^    E^0^ = −0.3419 V Ag^+^ + e^−^ → Ag     E^0^ = +0.7996 V Cu^2+^ + 2Ag^+^ → Cu^2+^ + 2Ag ↓  ∆E^0^ = +0.4577 V (4)

**Figure 17:**
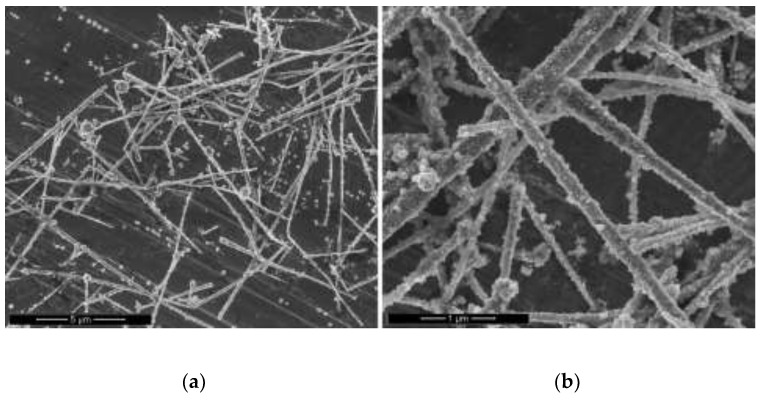
SEM images of Cu-Ag nanowires: (**a**) scale bar 5 µm; (**b**) 1 µm.

**Figure 18:**
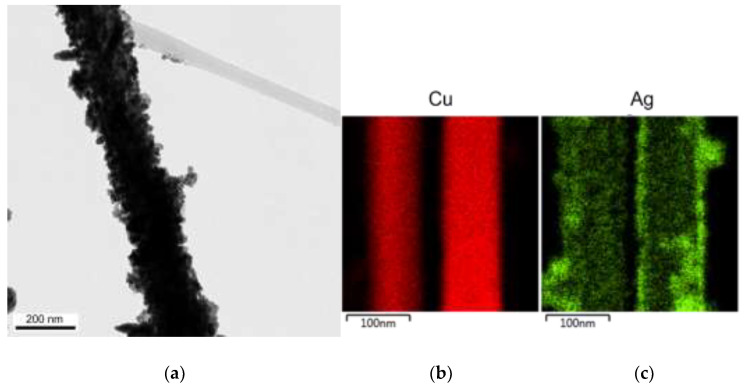
(**a**) TEM image of Cu-Ag nanowires; EDX mapping element (**b**) Cu; (**c**) Ag.

The redox reaction between copper and silver occurs spontaneously due to the positive difference redox potential (∆E^0^) of + 0.4577 V. Herein, DMSO_2_ could act as a silver reductant and a copper surfactant. When DMSO_2_ is added to the copper nanowire solution, copper atoms on the surface of the copper nanowires are oxidized to the copper ion state while protecting the surface of copper nanowires from reacting with other reagents. Upon introducing silver ions into the reaction solution, the surface of copper nanowires is coated by a redox reaction. As shown in Figure 17, silver is coated on the surfaces of copper nanowires. Figure 18a shows that the morphology of Cu-Ag nanowires includes slightly rough surfaces due to the formation of the silver shell. Figure 18b,c indicate the elemental composition of Cu-Ag nanowires obtained by EDX mapping. Figure 18b shows that copper is distributed throughout the nanowires, while Figure 18c shows that silver is strongly distributed on the surfaces of the copper nanowires. Fabricated Cu-Ag nanowires have a 90 nm copper core and a 12 nm thick silver shell. To prevent oxidation, the silver shell is required to be at least 5 nm thick, and a 15 nm thick silver shell protects against oxidation in humid conditions [33]. Therefore, a 12 nm thick silver shell not only could prevent the oxidation of copper nanowires, but also maintain suitable electrical or thermal conductivity that can compete with silver nanowires.

## 4. Conclusions

In conclusion, the morphology of copper nanowires was investigated by adding various concentrations of a reducing agent, a capping agent, a reagent, and a copper precursor, and various reaction temperatures. An excessive amount of N_2_H_4_ not only increased the diameter of copper nanowires but also caused an incomplete process of forming the dominant copper nanoparticles. At least 60 °C was required to prevent aggregations of copper seeds. However, as EDA performed actively at 70 °C, a smooth surface without aggregations of copper nanowires was observed. In addition, the amount of EDA was controlled to prevent the aggregation of copper seeds. An excessive amount of EDA erratically protected copper seeds and resulted in the irregular surface of copper nanowires. Therefore, it is required to interplay with NaOH and CuCl_2_. Copper nanowires synthesized under 9 M of NaOH, 50 µL of EDA, 17 mM of CuCl_2_, and 5.7 mM of N_2_H_4_ at a reaction temperature of 70 °C measured more than 18 µm in length and 25–45 nm in diameter without the aggregation of copper seeds. Cu-Ag nanowires were successfully fabricated by a new green method using DMSO_2_, which has not been reported previously for the fabrication of core–shell nanowires. DMSO_2_ acted as a copper surfactant and a silver reductant to initiate a redox reaction while protecting copper ions on the surfaces. The EDX mapping element exhibited the presence of a silver shell on the surface of copper nanowires. Fabricated Cu-Ag nanowires had a 90 nm copper core and a 12 nm thick silver shell. A silver shell 12 nm thick could prevent the oxidation of copper nanowires and maintain suitable electrical or thermal properties. In this study, we focused on the synthesis of copper nanowires depending on various conditions, and developed a new green method for the fabrication of Cu-Ag nanowires. For future work, phase characterization and the material properties, including electrical conductivity and thermal stability, should be studied.

## Figures and Tables

**Table 1:** Dimensions of copper nanowires synthesized with various amounts of N_2_H_4_.

N_2_H_4_ (µL)	Length (µm)	Diameter (nm)
15	4–13	250–550
30	4–15	450–750
240	Copper nanoparticles of the diameter of 800 nm	

**Table 2:** Dimensions of copper products synthesized at various reaction temperatures.

Reaction Temperature (°C)	Length (µm)	Diameter (nm)
40	more than 3.5	130–250
50	more than 5	130–280
60	more than 8	200–310
70	more than 5	210–260
80	more than 6	120–280
90	The oxidation of copper nanowires	

**Table 3:** Dimensions of copper nanowires synthesized under various amounts of EDA.

EDA	Length (µm)	Diameter (nm)
15	more than 4	150–220
50	more than 2	210–270
60	more than 6	130–170

**Table 4:** Dimensions of copper nanowires synthesized under the various concentrations of NaOH.

NaOH (M)	Length (µm)	Diameter (nm)
5	more than 3	130–250
7	more than 12	80–160
9	more than 18	25–45
12	more than 12	100–500

## Data Availability

The data presented in this study are available on request from the corresponding author.
